# Can modern diagnostics help in successful treatment of cervical necrotizing fasciitis?

**DOI:** 10.1007/s00405-017-4478-y

**Published:** 2017-02-10

**Authors:** Jerzy Kuczkowski, Tomasz Nowicki, Wojciech Brzoznowski

**Affiliations:** 1 Department of Otolaryngology, Medical University of Gdańsk (MUG), Smoluchowskiego 17, 80-214 Gdańsk, Poland; 2 II Department of Radiology, Medical University of Gdańsk (MUG), Gdańsk, Poland

To the Editor,

We have read with great interest the article “Cervical necrotizing fasciitis: descriptive, retrospective analysis of 59 cases treated at a single center” by Elander et al. [1]. The authors presented clinical analysis and management of 59 cases with cervical necrotizing fasciitis (CNF). We would like to make some comments concerning this problem, based on our experience and literature review [2–4]. CNF is a rare and potentially fatal infectious disease, which have occurred several times in our hospital (Medical University of Gdańsk). The treatment of CNF includes intravenous antibiotics, surgical debridement, and hyperbaric oxygen therapy. We would like to stress the role of modern bacteriological diagnostic methods which allow to confirm bacterial etiology in over 80% of the cases. Radiological tools (CT, MRI, and ultrasound) are useful in differentiating between locally limited cervical abscess and CNF. Such examinations are performed every day at the early phase of the disease [4]. We believe that CT is the tool of the most importance in observing the expansion of the inflammatory process in the cervix and thorax regions. The examination should be performed with contrast agent and should cover the neck from the basis of the scull to the sternoclavicular joints. In some cases, the chest CT is also necessary. In course of phlegmon, a non-enhanced CT can reveal thickening of soft tissues, asymmetry of neck structures, fluid collections, enlargement of lymph nodes, and the most importantly gas pockets formed by bacteria. In case of extension of the process into the subcutaneous tissue, fat stranding and irregular thickening of fascia can be seen. Contrast agent facilitates differentiation of fluid collections from inflammatory tissue thickening as involved tissue enhances. Moreover, contrast agent allows evaluation of potential vessel complications (arterial and venous occlusion, thrombosis and pseudoaneurysm formation). CT can facilitate diagnosis of CNF. Typical findings are asymmetrical thickening of superficial or/and profound fascia, fluid collections, and gas pockets. Sometimes, post-contrast enhancement of involved fascia can be observed. In the absence of typical radiological features in CT, MRI is helpful. MRI can provide early diagnosis and show the extend of inflammatory process. Involved soft tissues and fluid collections are hyperintens in T2-weighted images. In addition, contrast enhancement of soft tissues can be assessed in T1-weighted images. To assess the extension of inflammatory process into the mediastinum and pleural spaces, chest CT should be performed (Fig. 1a, b).

**Fig. 1:**
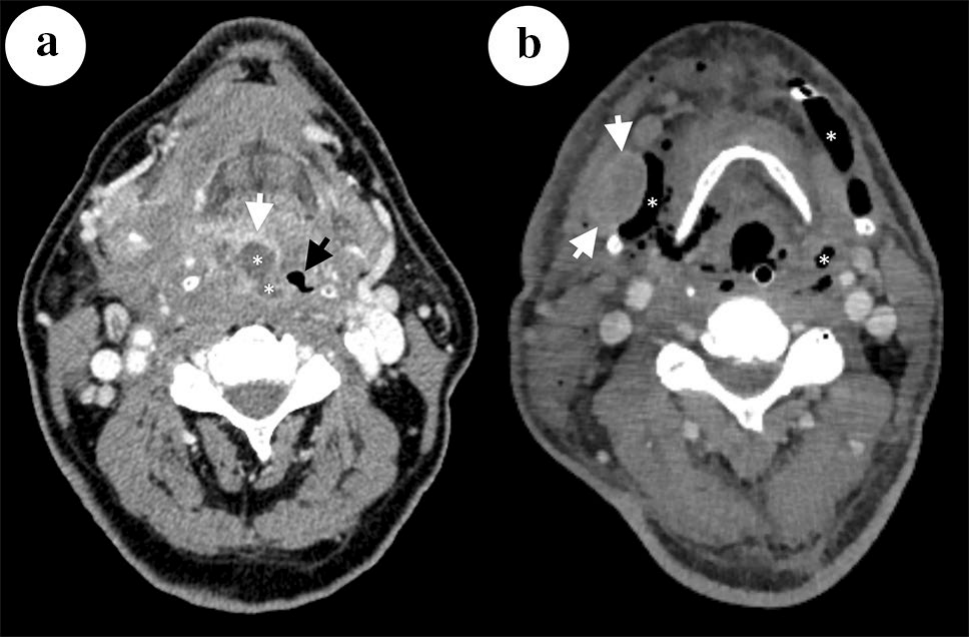
**a** Case of phlegmon of parapharyngeal space arising from right palatine tonsil in 74-year-old Caucasian male on axial CT image with contrast enhancement. Low attenuation fluid area (*asterisk*) with rim of enhancement (*white arrow*) and surrounding soft-tissue edema in right parapharyngeal space cause deviation of the pharynx and narrows its lumen (*black arrow*). **b** Case of phlegmon with gas formation arising from right palatine tonsil in 64-year-old Caucasian male on axial CT with contrast enhancement. Gas is visible between soft tissues (*asterisk*). Enlarged and heterogeneously enhancing lymph node in right submandibular region is present (*arrows*). In addition, subcutaneous tissue edema manifests as fat stranding

Ultrasound (US) examination plays minor role in diagnostics of cervical inflammatory processes. Although US can show thickening of soft tissues, fluid collections, gas pockets, increased echogenicity of the subcutaneous fatty tissue or intensified vascularization, it cannot assess profound neck structures or extension of the process into the mediastinum. Besides phlegmon and CNF, in differential diagnosis of cervical inflammatory processes, subcutaneous emphysema and cellulitis should be taken into consideration. We believe that modern radiological (CT and MRI) and bacteriological diagnostics allows to introduce promptly adequate treatment assessing the extent of surgical treatment, choice of antibiotic therapy, and use of hyperbaric oxygen therapy for full therapeutic success.

## References

[1] Elander J, Nekludov M, Larsson A (2016). Cervical necrotizing fasciitis: descriptive, retrospective analysis of 59 cases treated at a single center. Eur Arch Otorhinolaryngol.

[2] Krenk L, Nielsen H, Christensen M (2007). Necrotizing fasciitis in the head and neck region: an analysis of standard treatment effectiveness. Eur Arch Otorhinolaryngol.

[3] Wolf H, Rusan M, Lambertsen K, Ovesen T (2010). Necrotizing fasciitis of the head and neck. Head Neck.

[4] Wong CH, Wang YS (2005). The diagnosis of necrotizing fasciitis. Curr Opin Infect Dis.

